# Acupuncture for Spasticity after Stroke: A Systematic Review and Meta-Analysis of Randomized Controlled Trials

**DOI:** 10.1155/2015/870398

**Published:** 2015-01-05

**Authors:** Sung Min Lim, Junghee Yoo, Euiju Lee, Hyun Jung Kim, Seungwon Shin, Gajin Han, Hyeong Sik Ahn

**Affiliations:** ^1^Department of Motor & Cognition Rehabilitation, Korean National Rehabilitation Research Institute, 111 Gaorigil, Gangbuk-gu, Seoul 142-884, Republic of Korea; ^2^College of Korean Medicine, Kyung Hee University, 23 Kyungheedae-ro, Dongdaemun-gu, Seoul 130-872, Republic of Korea; ^3^Institute for Evidence-Based Medicine, Department of Preventive Medicine, College of Medicine, Korea University, 126-1 Anam-dong, Seongbuk-gu, Seoul 136-705, Republic of Korea

## Abstract

The aim of this systematic review was to determine how effective acupuncture or electroacupuncture (acupuncture with electrical stimulation) is in treating poststroke patients with spasticity. We searched publications in Medline, EMBASE, and the Cochrane Library in English, 19 accredited journals in Korean, and the China Integrated Knowledge Resources Database in Chinese through to July 30, 2013. We included randomized controlled trials (RCTs) with no language restrictions that compared the effects of acupuncture or electroacupuncture with usual care or placebo acupuncture. The two investigators assessed the risk of bias and statistical analyses were performed. Three RCTs in English, 1 in Korean, and 1 in Chinese were included. Assessments were performed primarily with the Modified Ashworth Scale (MAS). Meta-analysis showed that acupuncture or electroacupuncture significantly decreased spasticity after stroke. A subgroup analysis showed that acupuncture significantly decreased wrist, knee, and elbow spasticity in poststroke patients. Heterogeneity could be explained by the differences in control, acupoints, and the duration after stroke occurrence. In conclusion, acupuncture could be effective in decreasing spasticity after stroke, but long-term studies are needed to determine the longevity of treatment effects.

## 1. Introduction

Stroke is a disease that causes high rates of mortality and after-effects worldwide [1]. Spasticity is the most common poststroke complication [2], appearing in 20–40 percent of stroke survivors [3]. This not only restricts motor function [4] but also leads to deterioration in the quality of life for stroke patients [5].

Rehabilitation therapy for spasticity after stroke includes potentiating medication, neuromuscular electrical stimulation after botulinum toxin injections, restoring biomechanics through orthotics, stretching, functional electrical stimulation and treadmill exercises, and physical modalities, such as ultrasound, vibration, and thermotherapy, which should be adopted together and performed simultaneously to promote the improvement of motor function [5, 6]. However, more than half of stroke survivors with spasticity experience moderate to severe disabilities in spite of the conventional treatments [7].

The long-term management of spasticity is a financial burden to patients and their careers and also increases societal costs [1]. One study showed that the direct cost for 12-month stroke survivors with spasticity was Purchasing Power Parities US dollars (PPP$) 84,195, which is 385% higher than the PPP$ 21,842 for patients without spasticity [8].

These limitations have prompted researchers to look for new treatments to replace conventional treatments for poststroke spasticity and to consider the utility of acupuncture or electroacupuncture therapy.

Acupuncture therapy has been used to treat stroke patients for many years in Asian countries [1, 9] and also recently in the West [10]. Acupuncture stimulation sends signals to the central nerve system to release opioid peptides, resulting in an increase in the threshold of pain receptors [9, 11]. By controlling pain, acupuncture therapy helps muscles to relax and move more passively, resulting in an increase in rehabilitation [12].

A few recent systematic reviews have examined the effectiveness of acupuncture for poststroke rehabilitation [1, 13–17]. However, none have focused on spasticity after stroke. In an attempt to fill this gap, the current systematic review assesses how effective acupuncture or electroacupuncture is in treating poststroke patients with spasticity.

## 2. Methods

### 2.1. Identification of Eligible Trials

The search was performed without restriction with respect to language or year of publication. We searched Medline, EMBASE, and the Cochrane Central Register of Controlled Trials from database start through to July 30, 2013, combining medical subject headings and keyword terms for stroke, acupuncture, and muscle spasticity outcomes (Appendix A). For Korean publications, we manually searched 19 traditional medicine journals, which were accredited or chosen as candidates for accreditation by the National Research Foundation of Korea (http://www.nrf.re.kr) for relevant articles (Appendix B). The China Integrated Knowledge Resources Database (http://www.cnki.net) was also included to search related articles in Chinese. A hand search of relevant references from previous systematic reviews was conducted. Finally, we also searched an international database (https://www.clinicaltrials.gov/) for trial registrations to identify ongoing or recently completed trials.

### 2.2. Inclusion/Exclusion Criteria

Relevant clinical trials were manually selected based on the following criteria: (1) patients were diagnosed with stroke, (2) acupuncture was compared to placebo or other conventional therapy, and (3) the study was a randomized controlled trial (RCT). RCTs were included if acupuncture was used at acupoints as the sole treatment or as an adjunct to other treatments for spasticity after stroke.

Trials were excluded if study designs were not suitable to evaluate the effectiveness of acupuncture for spasticity after stroke, that is, any studies that (1) compared different types of acupuncture, (2) adopted complex treatment without specifying the sole effects of acupuncture, or (3) reported insufficient information.

### 2.3. Data Extraction

Two investigators (Sungmin Lim and Junghee Yoo) extracted data from each paper independently using a standardized data extraction form and reached consensus on all items. The extracted data included authors, published year, study design, patient characteristics, interventions, and main outcomes. We extracted the outcomes of pain, function, and symptom severity for all time points reported. When a given study reported more than one pain, function, or symptom severity measure, we gave preference primarily with Modified Ashworth Scale (MAS).

### 2.4. Assessment of Risk of Bias (ROB)

The two reviewers (Sungmin Lim and Junghee Yoo) independently assessed the methodological quality and the risk of bias of the included studies by means of the risk of bias tool in the Cochrane Handbook for Systematic Reviews of Interventions (version 5.0.2). This instrument consists of 6 domains and 8 items: random sequence generation; allocation concealment; blinding of participants, personnel, and outcomes; incomplete outcome data; selective outcome reporting; and the other source of bias which uses the following three categories (high risk, low risk, and unclear) to rank the evidence from research studies but is also appropriate for evaluating the methodological quality of RCTs. Disagreements between the reviewers were resolved by discussion and the input of a third reviewer (Euiju Lee). Publication bias was not a factor in the trials due to the limited number of studies.

### 2.5. Statistical Analysis

All statistical analyses were performed with the Reviewer Manager Software, version 5.0 (Cochrane Collaboration, Oxford, UK). As all outcomes were continuous variables, the mean difference with accompanying 95% confidence intervals was calculated. We assessed the clinical and methodological heterogeneities of the enrolled studies, according to which subgroup analysis was performed. The statistical heterogeneity in the subgroups was analyzed using the chi-square test (the significance level was *P* < 0.1). Statistical heterogeneity was considered to be significant when *I* ^2^ > 50%. Even when a low heterogeneity was detected, a random-effects model was applied, because the validity of tests of heterogeneity can be limited with a small number of component studies.

## 3. Results

### 3.1. General Characteristics of the Studies

**Figure 1:**
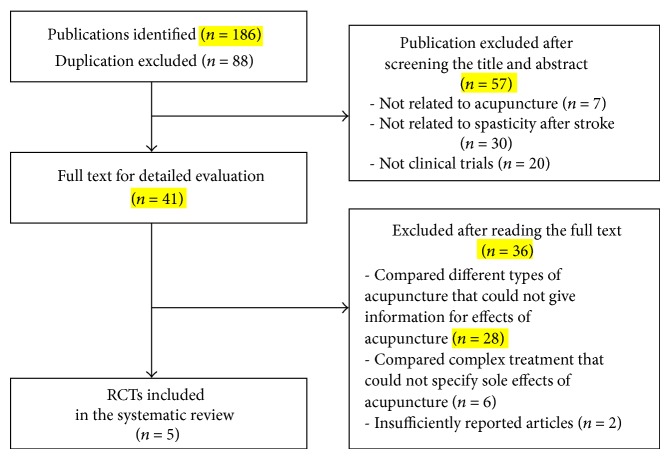
Flow chart of the trial selection process.

We identified 187 publications, of which 5 RCTs were finally included by the eligibility criteria (Figure 1). The excluded studies are listed in Appendix C. The articles included in the analysis are summarized in Table 1. The 5 articles were published from 2003 to 2012. Two of them originated from Korea [18, 19], 2 were from China [20, 21], and the other was from Germany [22]. The language of publication varied from English [19, 20, 22] to Chinese [21] or Korean [18].

Fink et al. [22] and Zhao et al. [20] studied the effectiveness of acupuncture on spasticity after stroke. The former study performed verum needle treatment on acupoints in the acupuncture group, which was compared with placebo needle treatment on nonacupoints in the control group. The latter study gave acupuncture therapy and standard therapy to the intervention group; the outcome was compared with that of a standard therapy group. Moon et al. [19], Lee et al. [23], and Zong [21] used electroacupuncture for participants with poststroke spasticity. In all of these studies, an electroacupuncture group with standard therapy was compared with a control group receiving only standard therapy.

The primary assessment tool for the 5 studies was the modified Ashworth scale (MAS). Four of the studies reported that acupuncture or electroacupuncture significantly reduced the spasticity after stroke.

### 3.2. Assessment of Risk of Bias (ROB)

**Figure 2:**
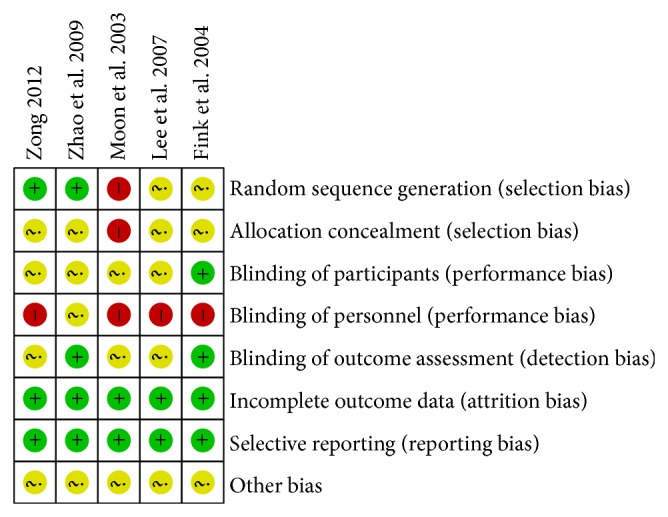
Assessment of risk of bias with selected studies.

The results of ROB were shown in Figure 2. Two RCTs [20, 21] had a low ROB with regard to adequate sequence generation; two [22, 23] had an unclear ROB; and one had a high ROB [19]. With regard to allocation concealment, four RCTs [20, 21] had an unclear ROB and one had a high ROB [19]. With regard to participant blinding, four RCTs [19–21, 23] had an unclear ROB and one had a low ROB [22]. With regard to personnel blinding, four RCTs [19, 21–23] had an unclear ROB and one had an unclear ROB [20]. With regard to assessor blinding, three RCTs [23, 24] had an unclear ROB [19, 21, 23] and two had a low ROB [20, 22]. All six RCTs had a low ROB in incomplete outcome data and selective outcome reporting. All six RCTs had an unclear ROB in other sources of bias.

### 3.3. Meta-Analysis of the Results

**Figure 3:**
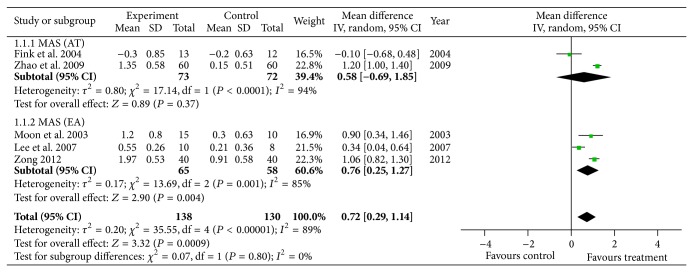
Meta-analysis of acupuncture for spasticity after stroke.

The pooled meta-analysis of the data showed a weighted mean difference of 0.72 and 95% confidence intervals of 0.29 to 1.14 on the MAS, indicating that acupuncture or electroacupuncture had a significant effect on decreasing poststroke spasticity (*P* < 0.001, *n* = 268; Figure 3).

In the subgroup analysis examining the types of acupuncture, electroacupuncture therapy significantly decreased spasticity after stroke (weighted mean difference of 0.76, 95% CI [0.25, 1.27], *P* = 0.004, *n* = 123), while acupuncture therapy showed slightly, but not significantly, decreased spasticity (weighted mean difference of 0.58, 95% CI [−0.69, 1.85], *P* = 0.37, *n* = 145; Figure 3).

**Figure 4:**
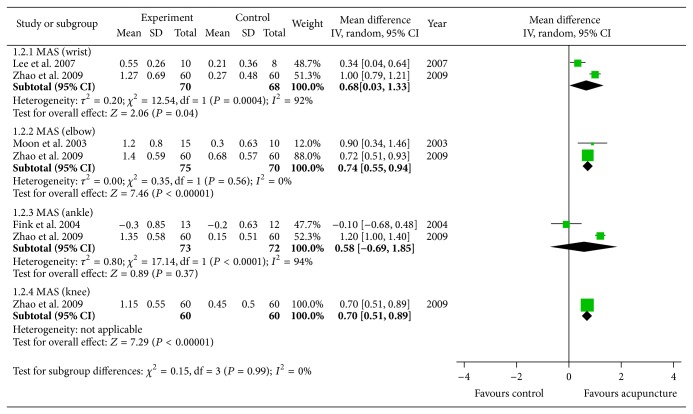
Meta-analysis of acupuncture for spasticity after stroke according to region.

The subgroup analysis based on the regions of spasticity revealed that acupuncture or electroacupuncture significantly reduced spasticity of wrists (weighted mean difference of 0.68, 95% CI [0.03, 1.33], *P* = 0.04, *n* = 138), knees (weighted mean difference of 0.70, 95% CI [0.51, 0.89], *P* < 0.001, *n* = 120), or elbows (weighted mean difference of 0.74, 95% CI [0.55, 0.94], *P* < 0.001, *n* = 145). There was some alleviation of spasticity of ankle region, but this was not statistically significant (weighted mean difference of 0.58, 95% CI [−0.69, 1.85], *P* = 0.37, *n* = 145; Figure 4).

## 4. Discussion

Our findings indicated that acupuncture or electroacupuncture therapy is effective in reducing the spasticity after stroke. Although the subgroup analyses indicated a nonsignificant effect of acupuncture on spasticity after stroke, this is partly an effect of the Fink et al.'s [22] study design. In this study, the control group did not receive any standard therapies, such as rehabilitation therapy, unlike the control groups from other studies using acupuncture interventions. If the data from Fink et al. [22] is excluded, the overall effect is 0.72 (weighted mean difference), which is a considerable elevation of grades on the MAS.

The difference between the mean value of 1.2 in Moon et al. [19] and the mean value of 0.55 in Lee et al. [23] results from the difference between the treatment groups of the two studies. Moon et al. [19] used electroacupuncture with routine acupuncture therapy, while Lee et al. [23] strictly excluded other standard therapies for the intervention group, demonstrating a more precise effect of acupuncture on poststroke spasticity.

Acupuncture or electroacupuncture is more effective in alleviating the spasticity of wrist, knee, and elbow after stroke. In this subgroup analysis, we were not able to estimate the effect on all of the regions, because only Zhao et al. [20] presented the MAS evaluation results of wrist, knee, elbow, and ankle. Since other studies measured only one region of spasticity, the total sum of data could be the cumulative effect on the different regions.

Caution should be exercised in including data from Fink et al. [22] in the overall synthesis and interpretation, for the following reasons: Firstly, the study has a different control group than the control groups in other studies. That is, other studies compared the standard therapy group with the acupuncture group; in contrast, Fink et al. [22] used placebo therapy (blunt needles on nonacupoints) compared with acupuncture therapy. Secondly, Fink et al. [22] acupunctured all over the body, including spasticity regions, unlike the other studies that used affected parts as the only regions for acupuncture. Thirdly, duration after stroke for participants included in the Fink et al. [22] study differs from that in the other studies. The duration after stroke onset was 1 to 17 months in most studies, while in Fink et al. [22] the patients were 65 months on average poststroke. It is very unlikely that patients whose stroke occurred 5 years ago would experience any alleviation of spasticity by the acupuncture treatments, because the after-effects of a stroke usually persist for 24 months. In other words, the timing of treatment is very important for spasticity after stroke. We could infer that acupuncture might be more effective in acute or semiacute stages of stroke than in the chronic stage. However, the small sample size prevents us from generalizing from this data.

In interpreting the results of this systematic review, there are several strengths to consider. Firstly, because acupuncture or electroacupuncture is effective for patients within 2 years after stroke onset, it should be adopted for the initial treatment of poststroke spasticity. Secondly, acupuncture therapy can promote the effectiveness of meaningful standard therapies to reduce spasticity. Thirdly, we aimed to identify all studies on the topic. The distorting effects of publication bias and location bias on systematic reviews are well documented [24–26]. In the present review, there were no restrictions on the review publication language, and a large number of different databases were searched. We are therefore confident that our search strategy located all relevant data on the subject.

However, certain limitations need to be considered as well. Above all, the number of RCTs included was small. This is because we restricted the inclusion criteria to the specific condition of spasticity after stroke and excluded publications studying the response within 24 hours after acupuncture therapy. Moreover, many of the reviewed studies were of low quality and had methodological shortcomings, such as an inadequate level of blinding. Although blinding of the therapists who applies acupuncture would be difficult, blinding of patients and other care providers, as well as outcome assessors, should be attempted to minimize the performance and assessment bias of trials. Lastly, there was no consistency in the regions of spasticity among studies. Only Zhao et al. [20] measured four regions of wrist, knee, elbow, and ankle, compared with the other studies, which treated only one of the regions.

Future trials should adhere to rigorous trial designs that are suitable for the research questions being addressed. To improve the trial design quality, level of performance, and the degree of reporting of clinical acupuncture trials, future researchers should follow not only the basic guidelines for reporting clinical trials such as the CONSORT statement [27], but also the STRICTA recommendations, which provide specific guidelines for the reporting of acupuncture trials [28]. Long-term studies are also needed to determine the longevity of treatment effects. Moreover, a cost analysis should also be considered.

## 5. Conclusions

Acupuncture or electroacupuncture could be effective in decreasing the spasticity after stroke, but long-term studies are needed to determine the longevity of treatment effects.

## Figures and Tables

**Table 1:** Summary of randomized controlled trials of acupuncture for spasticity after stroke.

Author (year) country	Sample size (analyzed)	Intervention group				Control group			Main outcomes (regions evaluated for MAS)
		*N* (analyzed)	Duration after stroke	Treatment	Regimen	*N* (analyzed)	Duration after stroke (mo/d)	Regimen	
Moon et al. (2003) [19] Korea	35 (35)	15 (15)	3.7 ± 3.7 mo	EA	8 sessions (EA, plus ST)	(A) 10 (10) (B) 10 (10)	(A) 2.7 ± 1.4 (B) 2.5 ± 1.8 mo	(A) ST (routine AT, exercises) (B) moxibustion, plus standard therapy	MAS (elbow)

Fink et al. (2004) [22] Germany	25 (25)	13 (13)	66.5 ± 50.2 mo	AT	8 sessions (AT)	12 (12)	64.2 ± 48.3 mo	Placebo AT	MAS (ankle) VAS, CGI, 2MWT, RMA, RMI, step length, cadence, mode of initial foot contact, goniometry, QOL measures

Lee et al. (2007) [23] Korea	20 (18)	10 (10)	NR	EA	10 sessions (EA, plus ST)	10 (8)	NR	ST (oral medication)	MAS (wrist) H/M ratio, FMA

Zhao et al. (2009) [20] China	131 (120)	67 (60)	16.34 ± 6.09 mo	AT	30 sessions (AT: surface projection zone of decussation of pyramid, standard therapy)	64 (60)	16.76 ± 6.89 mo	ST (oral medication, routine AT)	MAS (wrist, elbow, knee, ankle) FMA, BI, EMG

Zong (2012) [21] China	80 (80)	40 (40)	24.5 ± 5.88 days	EA	30 sessions (EA, plus ST)	40 (40)	23.6 ± 7.08 days	ST (oral medication, rehabilitation)	MAS (NR) FMA, MBI

EA: electroacupuncture, ST: standard therapy, MAS: Modified Ashworth Scale, AT: acupuncture therapy, VAS: visual analog scale, CGI: clinical global impressions, 2MWT: 2-minute walk test, RMA: Rivermead motor assessment, RMI: Rivermead mobility index, QOL: quality of life, NR: not reported, FMA: Fugi-Meyer motor function, BI: Barthel index, EMG: electromyography, and MBI: modified Barthel index.

## Appendices

### A. The Search Strings of Medline, EMBASE, and Cochrane Library

*Medline*

1. “Cerebrovascular Disorders”[tiab] OR “Brain Ischemia”[tiab] OR “Cerebral Hemorrhage”[tiab] OR “Stroke”[tiab] “Cerebrovascular”[tiab] OR “Cerebrovascular Disorder”[tiab] OR “cva”[tiab] 11511
2. (((“Cerebrovascular Disorders”[Mesh:NoExp]) OR “Brain Ischemia”[Mesh:NoExp]) OR “Cerebral Hemorrhage”[Mesh]) OR “Stroke”[Mesh] 157920
3. 1 or 2 161875
4. “acupuncture”[tiab] OR “electroacupuncture”[tiab] OR “electro-acupuncture”[tiab] 15154
5. ((“Acupuncture”[Mesh]) OR “Acupuncture Therapy”[Mesh:NoExp]) OR “Electroacupuncture”[Mesh] 13999
6. 4 or 5 17948
7. 3 and 6 562
8. 7/hsss 265
9. “Muscle Spasticity”[tiab] OR “Spasm”[tiab] OR “Muscle Hypertonia”[tiab] OR “Spasticity”[tiab] OR “Muscle tightness”[tiab] OR “Muscle stiffness”[tiab] OR “Muscle pull”[tiab] 25331
10. “Muscle Hypertonia”[Mesh]8687
11. 9 or 10 25331
12. 11 and 8 21.

*Embase*

1. ‘brain hemorrhage'/exp OR ‘brain infarction'/exp OR ‘brain ischemia'/exp OR ‘cerebrovascular accident'/exp 323266
2. ‘Cerebrovascular Disorders':ab,ti OR ‘Brain Ischemia':ab,ti OR ‘Cerebral Hemorrhage':ab,ti OR ‘Stroke':ab,ti ‘Cerebrovascular':ab,ti OR ‘Cerebrovascular Disorder':ab,ti OR ‘cva':ab,ti 16523
3. 1 or 2 327481
4. ‘acupuncture'/de OR ‘electroacupuncture'/exp 29411
5. ‘acupuncture':ab,ti OR ‘electroacupuncture':ab,ti OR ‘electro-acupuncture':ab,ti 21298
6. 4 or 5 31248
7. 3 and 6 1215
8. ‘clinical trial'/exp OR ‘randomized controlled trial':it OR ‘controlled clinical trial':it OR randomized:ab,ti OR placebo:ab,ti OR randomly:ab,ti OR trial:ab,ti OR groups:ab,ti NOT (‘animals'/exp NOT ‘humans'/exp) 2399837
9. 7 AND 8 499
10. ‘muscle hypertonia'/de OR ‘muscle rigidity'/exp OR ‘spasticity'/exp 24583
11. ‘Muscle Spasticity':ab,ti OR ‘Spasm':ab,ti OR ‘Muscle Hypertonia':ab,ti OR ‘Spasticity':ab,ti OR ‘Muscle tightness':ab,ti OR ‘Muscle stiffness':ab,ti OR ‘Muscle pull':ab,ti 26387
12. 10 or 11 42831
13. 12 and 9 38.

*Cochrane*

1. “Cerebrovascular Disorders” OR “Brain Ischemia” OR “Cerebral Hemorrhage” OR “Stroke” “Cerebrovascular” OR “Cerebrovascular Disorder” OR “cva”:ti,ab,kw (Word variations have been searched) 3878
2. MeSH descriptor: [Cerebrovascular Disorders] this term only 1365
3. MeSH descriptor: [Brain Ischemia] this term only 922
4. MeSH descriptor: [Cerebral Hemorrhage] explode all trees 619
5. MeSH descriptor: [Stroke] explode all trees 4440
6. 1–5/or 7503
7. “acupuncture” OR “electroacupuncture” OR “electro-acupuncture”:ti,ab,kw (Word variations have been searched) 6438
8. MeSH descriptor: [Acupuncture] explode all trees 134
9. MeSH descriptor: [Acupuncture Therapy] this term only 1785
10. MeSH descriptor: [Electroacupuncture] explode all trees 416
11. 7–10/or 6438
12. 6 and 11 234
13. “Muscle Spasticity” OR “Spasm” OR “Muscle Hypertonia” OR “Spasticity” OR “Muscle tightness” OR “Muscle stiffness” OR “Muscle pull”:ti,ab,kw (Word variations have been searched) 2420
14. MeSH descriptor: [Muscle Hypertonia] explode all trees 537
15. 13 or 14 2471
16. 15 and 12 16.

### B. Korean Journals for Oriental Medicine Included the Following

Korean Journal of Acupuncture,
Korean Journal of Oriental Physiology & Pathology,
The Korea Journal of Herbology,
Journal of Pharmacopuncture,
Korean Journal for Oriental Preventive Medical Society,
Journal of Korean Acupuncture and Moxibustion Medicine Society (The Acupuncture),
The Journal of Korean Oriental Internal Medicine,
The Journal of Oriental Obstetrics & Gynecology,
The Journal of Pediatrics of Korean Medicine,
The Korean Society of Oriental Neuropsychiatry,
The Journal of Korean Oriental Ophthalmology & Otorhinolaryngology & Dermatology,
The Journal of Korean Medical Classics,
Journal of Korean Medicine,
Journal of Sasang Constitutional Medicine,
Journal of Oriental Rehabilitation Medicine,
Journal of Korean Medicine Research in Daejeon University,
The Journal of The Korea Institute of Oriental Medical Diagnostics,
The Korean Journal of Oriental Medical Prescription,
The Journal of Korean Medical History.

### C. Excluded Studies (Author, Year, Title, Journal, Vol., Iss.)

#### C.1. Publication Excluded after Screening the Title and Abstract (*N* = 57)

*(a) Not Related to Acupuncture* (*N* = 7)

1. Lee, M. S., B. C. Shin, et al., 2010, Moxibustion for stroke rehabilitation: Systematic review, Stroke, 41, 4.
2. Li, H. F., J. H. Wang, et al., 2005, Application of motor relearning therapy in the early rehabilitation of stroke: A randomized controlled comparison, Chinese Journal of Clinical Rehabilitation, 9, 29.
3. Li, L. Z., 2004, Effect of zhongfeng erdai hurichun capsule combined with rehabilitation training on extremity spasticity of stroke, Chinese Journal of Clinical Rehabilitation, 8, 25.
4. Milanov, I. G., 1992, Flexor reflex for assessment of common interneurone activity in spasticity, Electromyography and Clinical Neurophysiology, 32, 12.
5. Teasell, R., S. Mehta, et al., 2012, Time to rethink long-term rehabilitation management of stroke patients, Topics in Stroke Rehabilitation, 19, 6.
6. Yan, T. and C. W. Hui-Chan, 2009, Transcutaneous electrical stimulation on acupuncture points improves muscle function in subjects after acute stroke: a randomized controlled trial, J Rehabil Med, 41, 5.
7. HU Nan, JIN Guoying, 2013, Curative Effect Observation on Improving Post-Stroke Spastic Mode, Liaoning Journal of Traditional Chinese Medicine, 2013, 5.

*(b) Not Related to Spasticity after Stroke* (*N* = 30)

1. Horng, M. S., 2005, Acupuncture shows no benifit over sham treatment for stroke rehabilitation, Journal of Clinical Outcomes Management, 12, 12.
2. Matsumoto, J., T. Aki, et al., 2012, Acupuncture treatment attenuates excitability of spinal motor neuron and spastic muscle overactivity in patients with persistent disturbance of consciousness following traumatic brain injury, Brain Injury, 26, 4-5.
3. Schiff, E., Y. H. Kim, et al., 2005, Vegetative states: An integrative approach, Integrative Medicine, 4, 1.
4. Singer, H. S., 2010, Treatment of tics and tourette syndrome, Current Treatment Options in Neurology, 12, 6.
5. Xu, J., 2007, Effect of post-stroke sensory disorders on the recovery processes of motor function and activity of daily living: A non-randomized synchronical controlled trial, Neural Regeneration Research, 2, 12.
6. Yen, H. L. and W. Chan, 2003, An east-west approach to the management of central post-stroke pain, Cerebrovascular Diseases, 16, 1.
7. Zhong, C. M., Q. F. Liu, et al., 2002, Effects of acupuncture and balance facilitation of muscular tension on the early rehabilitation of patients with stroke and hemiplegia, Chinese Journal of Clinical Rehabilitation, 6, 23.
8. Zhou, W. and L. P. Wang, 2005, Observation on therapeutic effect of abdominal acupuncture on spastic paralysis after cerebrovascular disorder, Zhongguo Zhen Jiu, 25, 11.
9. Dong, X. L., X. M. Feng, et al., 2003, Evaluation of the effects of acupuncture for the treatment of hemipleyis in patients with stroke by Fugl-Meyer, Chinese Journal of Clinical Rehabilitation, 7, 19.
10. Gong, W., T. Zhang, et al., 2009, Electro-acupuncture at Zusanli (ST 36) to improve lower extremity motor function in sensory disturbance patients with cerebral stroke: A randomized controlled study of 240 cases, Neural Regeneration Research, 4, 11.
11. Liu, W., M. Mukherjee, et al., 2008, Electroacupuncture may help motor recovery in chronic stroke survivors: a pilot study, J Rehabil Res Dev, 45, 4.
12. Schaechter, J. D., B. D. Connell, et al., 2007, Correlated change in upper limb function and motor cortex activation after verum and sham acupuncture in patients with chronic stroke, Journal of Alternative and Complementary Medicine, 13, 5.
13. Wayne, P. M., D. E. Krebs, et al., 2005, Acupuncture for upper-extremity rehabilitation in chronic stroke: A randomized sham-controlled study, Archives of Physical Medicine and Rehabilitation, 86, 12.
14. Wu, Y., 2008, Effects of electroacupuncture at the nerve trunk for treatment of apoplectic hemiplegia at the spastic stage, Journal of Traditional Chinese Medicine, 28, 3.
15. Yue, Z. H., L. Li, et al., 2012, Comparative study on effects between electroacupuncture and acupuncture for spastic paralysis after stroke, Zhongguo zhen jiu, 32, 7.
16. LANG Jian-ying, ZHUANG Li-xing, HE Jun, JIA Chao, ZHOU Zhao-hui, KE Li-ping, 2013, Randomized Controlled Study on Jin's Three Needle Therapy on Spastic Hemiplesia after Ischemic Stroke, Shanghai Journal of Acupuncture and Moxibustion, 2013, 6.
17. HE Yang-yang, HU Ka-ming, GUO Yao-guang, LAO Xiang-ting, ZAI Xi, CHI Yang-feng, YANG Jin, 2013, Clinical Observations on Liver-reinforcing and Spasm-relieving Acupuncture as Main Treatment for Post-stroke Neurological Deficits, Shanghai Journal of Acupuncture and Moxibustion, 2013, 4.
18. SU Su, SUN Yuan-zheng, 2013, Clinical Observation on Needling Antagonistic Muscle plus Rehabilitation for Lower-limb Hypertonia after Stroke, Shanghai Journal of Acupuncture and Moxibustion, 2013, 6.
19. NI Huan-huan, CUI Xiao, HU Yong-shan, WU Yi, HUANG De-quan, QU Pei-yu, WANG Jun, WU Ji, SHI Jun-chao, 2012, Therapeutic Observation on Acupuncture plus Rehabilitation for Upper-limb Spasticity after Cerebral Apoplexy, Shanghai Journal of Acupuncture and Moxibustion, 2012, 11.
20. Jiang Zaiyi, Han Guodong, 2011, Xuesaitong injection point injection of spastic limb spasticity after stroke improved clinical efficacy, Guiding Journal of Traditional Chinese Medicine and Pharmacy, 2011, 11.
21. HAN Shu-kai, ZHANG Bao-chang, ZUO Yong-fa, WEN Xiao-yu, 2010, Observations on the Efficacy of Muscle-region Alignment Needling plus Skin Acupuncture in Treating Post-stroke Upper Limb Spasticity, Shanghai Journal of Acupuncture and Moxibustion, 2010, 5.
22. 杨庆红, 孔妍 and 唐强, 2010, 针刺结合康复技术治疗脑梗死后痉挛 68 例 (no English Abstract), Journal of Clinical Acupuncture and Moxibustion, 2010, 11.
23. YUE Zeng-hui, LI Liang, YE Yu, JIANG Jing-ming, LI Xiang-rong, 2010, Effect of acupuncture on content of serum amino acid in spasticity paralysis after stroke, Journal of  Traditional Chinese Medicine University of Hunan, 2010, 9.
24. WU Ping, LIANG Fan-rong, LI Ying, JIN Rong-jiang, HU Ka-ming, TIAN Wei-wei, LUO Lun, YUAN Xiu-li, 2010, Research of efficacy assessment of post-stroke hemiplegic spasticity treated with acupuncture and rehabilitation: a multi-centre randomized controlled trial, World Journal of Acupuncture-Moxibustion, 2010, 2.
25. JIN Rong-jiang, ZHU Tian-ming, WANG Qian, 2010, Clinical Observation on Spastic Paralysis Caused by Cerebral Infarction Treated by Electric Stimulation on Acupuncture of Antagonistic Muscles and Facilitation Techniques, Journal of Chengdu University of Traditional Chinese Medicine, 2010, 3.
26. WU Ping, LIANG Fan-rong, YANG Ling, LUO Lun, LI An-hong, 2009, Effect of Acupuncture for Neurological Functional Deficit in the Spasticity Paitients after Stroke, Journal of Clinical Acupuncture and Moxibustion, 2009, 6.
27. 任亚锋, 2008, 针刺结合康复疗法治疗卒中后痉挛的临床研究 (no English Abstract), 中国医药指南, 2008, 23.
28. LI Yi, LI Yu-feng, PAN Fu-qiong, et al., 2008, Effect of Acupuncture Cooperating with Bobath Approach on Spasticity after Stroke, Chinese Journal of Rehabilitation Theory and Practice, 2008, 11.
29. MI Jian-ping, ZHANG Zhong-cheng, 2006, Clinical Observation on Therapeutic Effect of Yin-meridian Electroacupuncture in Reducing Muscular Tension of Limbs in Apoplectic Hemiplegia, Journal of Acupuncture and Tuina Science, 2006, 3.
30. Eom Jaeyong, Won Seunghwan, Kwon Kirok, Lee Hyangsook, 2006, Comparative study of Acupuncture, Bee Venom Acupuncture and Bee Venom Herbal Acupuncture on the treatment of Post-stroke Hemiplegic Shoulder Pain, Journal of Pharmacopuncture, 9, 1.

*(c) Not Clinical Trials* (*N* = 20)

1. Bhogal, S. K., R. W. Teasell, et al., 2003, Quality of the stroke rehabilitation research, Topics in Stroke Rehabilitation, 10, 1.
2. Bond, H., 2004, September FACT looks at acupuncture, Pharmaceutical Journal, 273, 7316.
3. Fragoso, A. P. and S. Ferreira Ade, 2012, Evaluation of the immediate effects of manual acupuncture on brachial bicep muscle function in healthy individuals and post stroke patients: a study protocol of a parallel-group randomized clinical trial, Zhong Xi Yi Jie He Xue Bao, 10, 3.
4. Fu, Q. H., J. Pei, et al., 2012, Acupuncture treatment programs for post-stroke motor rehabilitation in community hospitals: study protocol of a multicenter, randomized, controlled trial, Zhong Xi Yi Jie He Xue Bao, 10, 5.
5. Quinn, T. J., S. Paolucci, et al., 2009, Evidence-based stroke rehabilitation: An expanded guidance document from the European Stroke Organisation (ESO) guidelines for management of ischaemic stroke and transient ischaemic attack 2008, Journal of Rehabilitation Medicine, 41, 2.
6. Lee, S. H., Y. O. Wong, et al., 2012, Acupuncture in managing of spasticity of post stroke survivor: A systematic review, Stroke, 43, 2.
7. WU Li, ZHANG Han-dan, WU Xi, DU Chen, LAN Ying, 2013, Investigation on Prescription Rules of Acupuncture and Moxibustion for Post-stroke Muscular Spasm, Journal of Clinical Acupuncture and Moxibustion, 2013, 4.
8. ZHANG Jia-qi, HAI Ying, 2011, Spastic hemiplegia dialectic of Acupuncture, Journal of Practical Traditional Chinese Internal Medicine, 2011, 7.
9. YE Xiao-qin, XIE Yan-ming, JI Lin-hong, 2011, The Ideas and Methods of Acupucture Treatment of Spasm after Stroke and the Investigation of Its Application in Rehabilitation Training Robot, Chinese Archives of Traditional Chinese Medicine, 2011, 7.
10. ZENG Deng-hui, ZHOU Wen-qiang, 2010, The Research Advancement of Limb Spasm after Brain Stroke by Acupuncture Treatment, Journal of Clinical Acupuncture and Moxibustion, 2010, 12.
11. HE Xi-ping, 2009, Advancement of Studies with Acupuncture and Moxibustion in Treatment of Muscle Spasm Because of Cerebral Accident, Journal of Liaoning University of Traditional Chinese Medicine, 2009, 11.
12. CHEN Ying, 2008, Clinical Observation of Treating Spasm after Apoplexy with Head Acupuncture Integrating Musculature Acupuncture Methods, Chinese Archives of Traditional Chinese Medicine, 2008, 5.
13. HE Xiao-hua, 2008, Clinical Observation of Acupuncture in Treating Upper Limb Spasticity After Stroke, Shanghai Journal of Traditional Chinese Medicine, 2008, 12.
14. LI Jia, HE Jing, 2008, General situation of acupuncture and moxibustion therapy on Limb Spasm due to stroke, Journal of Clinical Acupuncture and Moxibustion, 2008, 3.
15. HE Yong, JIN Rong-jiang, 2006, Advance in Treatment of Limb Spasm of Hemiplegic Patients (review), Chinese Journal of Rehabilitation Theory and Practice, 2006, 10.
16. MI Jian ping, ZHANG Zhong cheng, 2003, Clinical Observations on the Reducing Effect of Yin Meridian Electroacupuncture on Muscular Tension of Limb in Apoplectic Hemiplegia, Shanghai Journal of Acupuncture and Moxibustion, 2003, 10.
17. Park, Young-chul, Chae, JIn-seok, Eom, Jae-yong, Son Sung-se, Choe Ick-seon, 2003, Thermographic Study on the Effects of Deep Acupuncture at Hakok (L14) in Cerebrovascular Hemiplegia, The Journal Of Korean Acupuncture & Moxibustion Medicine Society, 20, 4.
18. Seo Byungkwan, Baek Yonghyeon, Park Dongsuk, 2010, A Systematic Review of Clinical Trials on Acupuncture on the Post-stroke Muscle Spasticity, The Journal Of Korean Acupuncture & Moxibustion Medicine Society, 27, 6.
19. Kang Sung Keel, Kim Yong Suk, 2010, EFFECT OF ORIENTAL MEDICINE ON THE RECOVERY FROM ACUTE STROKE, Journal of KyungHee Oriental Medicine College, 19, 1.
20. Kim Jihye, Kwak Hyunyoung, Kwon Youjung, Seon Jongin, Lee Ungin, Nam Dongwoo, Choi Doyoung, Lee Jaedong, 2011, A Case Report on the Effect of Electroacupuncture at LI_15_ and TE_14_ for the Treatment of Shoulder Pain in Post-stroke Hemiplegia Patients, The Journal Of Korean Acupuncture & Moxibustion Medicine Society, 28, 6.

#### C.2. Excluded after Reading the Full Text (*N* = 36)

*(a) Compared Different Types of Acupuncture That Could Not Give Information for Effects of Acupuncture (N* = 28)

1. Fan, L., X. P. Zhu, et al., 2005, Acupuncture applied on the deltoid muscle and musculus triceps brachii for upper limb muscle spasm in patients with cerebral infarction: Comparison with the acupuncture at yang channel point, Chinese Journal of Clinical Rehabilitation, 9, 25.
2. Hai, Y. and X. Yu, 2007, Observation on therapeutic effect of acupuncture on spastic dyskinesia due to stroke, Zhongguo zhen jiu, 27, 10.
3. Lou, B. D., W. Zhang, et al., 2010, Clinical evaluation on balanced muscular tension needling method for improving disabled function of stroke patients with spastic paralysis, Zhongguo zhen jiu, 30, 2.
4. Wang, L. C., Z. Y. Wang, et al., 2008, Comparative study on effects of different acupuncture manipulation methods at neiguan (PC 6) on hand spasm in the patient of stroke, Zhongguo zhen jiu, 28, 7.
5. Wang, L. P., W. Zhou, et al., 2007, Assessment and investigation of therapeutic effects of different needling methods for treatment of spastic state of post-cerebrovascular disease, Zhongguo zhen jiu, 27, 5.
6. Wang, X. B., J. Chen, et al., 2011, Effect of electroacupuncture in different frequencies on electromyography and ambulation in stroke patients with lower-extremity spasticity: a randomized controlled study, Zhongguo zhen jiu, 31, 7.
7. Yue, Z. H., 2005, Evaluation of therapeutic effect of muscle region needling for post-stroke spasticity a randomized controlled trial, Chinese Journal of Clinical Rehabilitation, 9, 9.
8. 刘 保 庚, 2012, 针刺足三阳经穴对中风偏瘫下肢痉挛状态治疗作用的对比研究 (no English Abstract), 针灸临床杂志, 2012, 2.
9. LI Fang-ling, 2012, Observations on the Efficacy of Antagonistic Muscle Acupuncture plus Rehabilitation Training in Treating Post-stroke Spastic Hemiplegia, Shanghai Journal of Acupuncture and Moxibustion, 2012, 11.
10. 肖 源 (no English Author Name), 2011, Treating 35 cases of stroke spasticity of ankle dysfunction by electro-acupuncture combined with rehabilitation, Clinical Journal of Chinese Medicine, 2011, 21.
11. WANG Wenchun, SONG Qingjun, WANG Qian, et al., 2011, Randomized controlled trial of treatment of hemiplegia and spasticity between electro-acupuncture at antagonistic muscular motor points and at acupoints in stroke patients, Chinese Journal of Rehabilitation Medicine, 2011, 5.
12. DAI Zhen, 2010, The Clinical Effect of Acupuncture Reinforcement and Reduction and Rehabilitation in Treating Muscular Spasm Caused By Apoplexy, Journal of Practical Traditional Chinese Internal Medicine, 2010, 9.
13. JIN Ze, WANG Lin-jing, 2010, Clinical Observations on Acupuncture at Huatuojiaji Points for the Treatment of Post-stroke Hemiplegic Spasticity, Shanghai Journal of Acupuncture and Moxibustion, 2010, 6.
14. 齐 欢 (no English Author Name), 2009, Clinical Observation on Electro-Acupuncture at Jiaji for the Treatment of upper Extremity Spasticity for Stroke Patients, 成都中医药大学学报 (no English Journal Title), 2009, —.
15. CAO Chen-hong, LIAN Yu-lin No, 2008, Observations on the curative effect of motor acupuncture on upper limb spasm after stroke, Journal of Clinical Acupuncture and Moxibustion, 2008, 8.
16. WANG Xiang-bin, HE Jian, LI Tian-jiao, et al., 2008, Comparison of Surface Electromyography between High and Low Frequency Electro-acupuncture Therapy in Stroke Patients with Spasticity on Lower Limbs, Journal of Fujian University of Traditional Chinese Medicine, 2008, 6.
17. HAI Ying, YU Xiao, 2007, Observation on therapeutic effect of acupuncture on spastic dyskinesia due to stroke, Chinese Acupuncture & Moxibustion, 2007, 10.
18. WANG Li-ping, ZHOU Wei, ZHANG Shu-yuan, 2007, Investigation and Evaluation of the Efficacy of Scalp and Body Acupuncture for Treating Spastic Paralysis after Cerebrovascular Diseases, Shanghai Journal of Acupuncture and Moxibustion, 2007, 7.
19. ZHANG Wen-dong, CHEN Xin-sheng, HAN Wei, CHEN Hao, YU Hong-wu, ZHOU Ting, 2005, Clinical Study on Treatment of Post-apoplectic Limb Spasm by Acupuncturing Du Meridian as A Main Therapy, Shanghai Journal of Acupuncture and Moxibustion, 2005, 5.
20. Yong-Suk Kim, 2000, Antispastic Effects of Electroacupuncture, TENS and NMES in Stroke Patient, The Journal Of Korean Acupuncture & Moxibustion Medicine Society, 17, 2.
21. Woo-Jin Sim, Suk-Hee Jung, Sun-Soo Kim, Hyun-Dae Shin, Jong-Soo Lee, 2003, Which is More Effective for Elbow Spasticity after Stroke, the Electroacupuncture on Yin or Yang Meridians?, Journal of oriental rehabilitation medicine, 13, 1.
22. Min-Beom Kim, Hyun-Dae Shin, Sung-Soo Kim, 2005, The Influences of Electroacupuncture at Interosseous Muscle for Hand Function in Hemiplegic Patients after Stoke, Journal of oriental rehabilitation medicine, 15, 4.
23. Kim Jayoung, Jeong Seonmee, Park Chankyu, Min Eunkyung, Wang Tehchung, 2008, The Clinical Effectiveness of Acupuncture at* Palsa(BaXie)* for Hand Function in Hemiparetic Patients after Stroke, The Journal Of Korean Acupuncture & Moxibustion Medicine Society, 25, 5.
24. You-Suk Youn, Woo-Jin Shim, Jong-Soo Lee, Sung-Soo Kim, 2004, The Effectiveness of Electroacupuncture Stimulateously Muscle Pattern Exercise for Gait Rehabilitation after Stroke, Journal of oriental rehabilitation medicine, 14, 1.
25. Cho Tai-sung, Son In-seok, Kim Cheol-hong, Seo Jung-chul, Youn Hyun-min, Jang Kyung-jeon, Song Choon-ho, Ahn Chang-beohm, 2002, Effects of Added Tong's Acupuncture on NIH Stroke ScaIe and MBI in Stroke Patients, The Journal Of Korean Acupuncture & Moxibustion Medicine Society, 19, 5.
26. Taesung Cho, Insuk Son, Inbeohm Park, Sangwoo Kim, Jungchul Seo, Hyounmin Youn, Kyungjeon Jang, Choonho Song, Changbeohm Ahn, 2003, Effects of Scalp Acupuncture on Short-term NIHSS and MBI in Stroke Patients, Journal of Korean Oriental Medicine, 24, 1.
27. Park Saewook, Lee Mingoo, Lee Sunwoo, Kang Baekgyu, Son Jiwoo, Park Sangmoo, Lee In, Moon Byungsoon, 2006, The Effect of Electroacupuncture by Yin and Yang meridians on Leg Spasticity of Stroke Patients, The Journal of Korean Oriental Internal Medicine, 27, 2.
28. Jungchul Seo, Yonghyeon Baek, Tonghyun Nam, Dongmin Seo, Hyunjong Lee, Jiyoung Ha, Hyunsu Woo, Jaedong Lee, 2001, Effects of Contralateral Both Side Acupuncture on NIH Scale in Stroke Patients, Journal of Korean Oriental Medicine, 22, 3.

*(b) Compared Complex Treatment That Could Not Specify Sole Effects of Acupuncture (N* = 6)

1. Qu, Y., M. Sheng, et al., 2003, Rehabilitation therapy centralized on facilitation and acupuncture on upper extremities spasm after stroke, Chinese Journal of Clinical Rehabilitation, 7, 1.
2. Wang, D. S., S. Wang, et al., 2008, Clinical observation on acupuncture with pushing manipulation for treatment of finger flexion in the patient of poststroke, Zhongguo zhen jiu, 28, 4.
3. Zeng, H. W., 2005, Assorted acupuncture and moxibustion combined with self-made Chinese herb recipe for improving the limbs spasticity and motor function in patients with stroke, Chinese Journal of Clinical Rehabilitation, 9, 17.
4. HA Jing, YE Ga-xi, JIA Hong-yun, HUANG Yin-lan, WAN Ming-yu, CAO Li-cui, WANG Lei, 2013, A clinical study of antagonistic muscle groups of acupoints of acupuncture treatment of hemiplegic limb spasticity after stroke, Lishizhen Medicine and Materia Medica Research, 2013, 2.
5. Zhu Fenglei, 2006, The Study of the Acupuncture and Low Frequency Electra Acupuncture with Rehabilitation Treating hemiplegia spasticity in stroke, Liaoning Journal of Traditional Chinese Medicine, 2006, 7.
6. Soonhyun Ryu, Kyungsup Lee, Taekyung Kim, Yosub Choi, Sangpil Yun, Jongchurl Jang, Sangkwan Moon, Changnam Ko, Kiho Cho, Youngsuk Kim, Hyungsup Bae, 2002, Effects of Electroacupuncture on the Hemiplegic Upper Extremity after Stroke, Journal of Korean Oriental Medicine, 23, 2.

*(c) Insufficiently Reported Articles (N* = 2)

1. Mukherjee, M., L. K. McPeak, et al., 2007, The Effect of Electro-Acupuncture on Spasticity of the Wrist Joint in Chronic Stroke Survivors, Archives of Physical Medicine and Rehabilitation, 88, 2.
2. Zhang, Z. M., C. L. Feng, et al., 2008, Observation on clinical therapeutic effect of acupuncture on upper limb spasticity in the patient of poststroke, Zhongguo zhen jiu, 28, 4.
