# Corrigendum to “Acupuncture for Spasticity after Stroke: A Systematic Review and Meta-Analysis of Randomized Controlled Trials”

**DOI:** 10.1155/2017/7324857

**Published:** 2017-04-05

**Authors:** Sung Min Lim, Junghee Yoo, Euiju Lee, Hyun Jung Kim, Seungwon Shin, Gajin Han, Hyeong Sik Ahn

**Affiliations:** ^1^Department of Motor & Cognition Rehabilitation, Korean National Rehabilitation Research Institute, 111 Gaorigil, Gangbuk-gu, Seoul 142-884, Republic of Korea; ^2^College of Korean Medicine, Kyung Hee University, 23 Kyungheedae-ro, Dongdaemun-gu, Seoul 130-872, Republic of Korea; ^3^Institute for Evidence-Based Medicine, Department of Preventive Medicine, College of Medicine, Korea University, 126-1 Anam-dong, Seongbuk-gu, Seoul 136-705, Republic of Korea; ^4^Department of Clinical Korean Medicine, Graduate School, Kyung Hee University, 26 Kyungheedae-ro, Dongdaemun-gu, Seoul 02447, Republic of Korea

In the article titled “Acupuncture for Spasticity after Stroke: A Systematic Review and Meta-Analysis of Randomized Controlled Trials” [1], the fifth author's affiliation should be changed to “Department of Clinical Korean Medicine, Graduate School, Kyung Hee University, 26 Kyungheedae-ro, Dongdaemun-gu, Seoul 02447, Republic of Korea.” The corrected list of affiliations is shown above.
